# Mobile Critical Care in Resource-Limited Settings: An Unmet Need

**DOI:** 10.5334/aogh.4506

**Published:** 2024-09-19

**Authors:** Varun U. Shetty

**Affiliations:** 1 Intensivist, Cleveland Clinic, Clinical Assistant Professor, Case Western Reserve University Lerner College of Medicine, OH, USA

**Keywords:** Global health critical care, mobile ICU, mobile critical care unit, critical care outreach, critical care, intensive care, acute care, essential care, emergency medicine

## Abstract

Care of the critically ill in resource-limited areas, inside or outside the intensive care unit (ICU), is indispensable. Murthy and Adhikari noted that about 70% of patients in low-middle income (LMIC) areas could benefit from good critical care. Many patients in resource-limited settings still die before getting to the hospital. Investing in capacity building by strengthening and expanding ICU capability and training intensivists, critical care nurses, respiratory therapists, and other ICU staff is essential, but this process will take years. Also, having advanced healthcare facilities that are still far from remote areas will not do much to alleviate distance and mode of transportation as barriers to achieving good critical care. This paper discusses the importance of mobile critical care units (MCCUs) in supporting and enhancing existing emergency medical systems. MCCUs will be crucial in addressing critical delays in transportation and time to receive appropriate lifesaving critical care in remote areas. They are incredibly versatile and could be used to transfer severely ill patients to a higher level of care from the field, safely transfer critically ill patients between hospitals, and, sometimes, almost more importantly, provide standalone short-term critical care in regions where ICUs might be absent or immediately inaccessible. MCCUs should not be used as a substitute for primary care or to bypass readily available services at local healthcare centers. It is essential to rethink the traditional paradigm of ‘prehospital care’ and ‘hospital care’ and focus on improving the care of critically ill patients from the field to the hospital.

## Introduction

Care of the critically ill in resource-limited areas, inside or outside the intensive care unit (ICU), is indispensable. Murthy and Adhikari noted that about 70% of patients in low-middle income (LMIC) areas could benefit from good critical care [1]. Many patients in resource-limited settings still die before getting to the hospital [2]. This paper discusses the importance of mobile critical care units (MCCUs) in supporting and enhancing existing healthcare delivery systems.

## Background and Scope of the Problem

In resource-limited settings, ICU care often differs from the prolonged end-of-life ICU care common in high-income countries. Critically ill patients can present at all levels of health care, and managing critical illness occurs as much outside of a dedicated ICU as it does within. Acute critical illnesses, such as sepsis, diarrheal illnesses, strokes, and ischemic heart disease, are some of the leading causes of death in resource-limited settings [3] and often need only short-term ICU support. For example, between 81,000–130,000 people die each year from snakebites [4, 5]. The mortality burden from snakebites is enormous, yet it is easily treatable with timely anti-venom and adequate critical care support. Patients often die before getting to the hospital, in part because of a fragmented system of prehospital care [5–7]. Frequently, patients are left to their own devices to find transport for long distances to find lifesaving treatment using taxis or rickshaws [8, 9].

In high-income countries, evidence supports a high-quality pre-hospital chain of care [10]. Yet, less than one percent of regions in LMICs have access to emergency medical transport services [11]. Referral systems in resource-limited areas are often not well organized, and a lack of a coordinated ambulance network has been known to increase delays in care [12]. This dearth of prehospital services cuts across various medical problems. For example, maternal mortality remains a significant problem in low and low-middle-income countries, with hemorrhage being one of the leading causes of death [13]. This can significantly benefit from early intervention. Even a three-hour delay in getting to the hospital can increase neonatal mortality [14]. About 90% of all trauma-related mortality occurs in low- and low-middle-income countries, [15] and the majority of trauma deaths happen in the prehospital setting [16]. In trauma literature, prehospital time has been linked to higher mortality, albeit inconsistently. However, what appears to be most useful is the skillset of the transporting practitioners, such as the ability to manage chest trauma, advanced airway placement, etc., that would be available in mobile critical care units [17, 18]. This is also evident in the literature showing how prehospital care can reduce mortality independent of the destination facility [19].

Hospitals in LMICs are often significantly strained and under-resourced. There is a significant shortage of ICU beds and staff in resource-limited settings, with 0.5–2.5 ICU beds per 100,000 people compared to the 5–30 beds per 100,000 seen in high-income countries [20]. The widespread staffing shortages in these countries further lead to an increased mortality burden [21].

The massive, unappreciated problem of the lack of infrastructure to manage critically ill people crystallizes episodically when there is a significant pandemic such as COVID-19. During these devastating and desperate situations, governments and healthcare organizations find themselves scrambling to develop infrastructure in a short period.

Appropriate short-term ICU interventions across the spectrum of critical illness can be lifesaving and put many back into the country’s workforce. Still, they are not always available to patients in LMICs [1, 22]. Investing in capacity building by strengthening and expanding ICU capability and training intensivists, critical care nurses, respiratory therapists, and other ICU staff is essential, but this process will take years. Also, having advanced healthcare facilities that are still far from remote areas will not do much to alleviate distance and mode of transportation as barriers to achieving good critical care. A well-equipped, reliable emergency/critical care service system is essential to provide critical care to patients and reduce barriers to accessing healthcare, disability, and mortality.

## Mobile Critical Care Units as a Potential Solution

**Figure 1:**
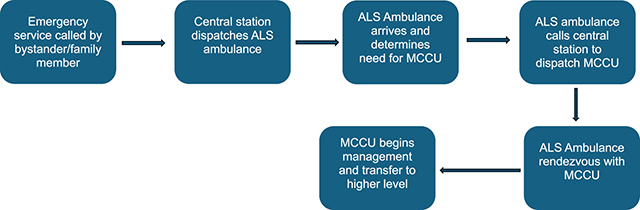
Example of mobile critical care unit workflow.

I propose an enhanced system of emergency medical services that utilizes MCCUs not just for interhospital transfers but also for providing care on the field that would be too complex for an ALS ambulance. The MCCUs would enhance already existing emergency medical services (Figure 1).

Evidence of efficacy: Critical care exists on a continuum involving prehospital care, ED/ICU, and transitions of care between prehospital and various hospital settings [11]. The mobile ICU could fulfill many of these crucial roles in low-resource settings. MCCUs are frequently used in high-income countries for the interhospital transfer of critically ill patients or transport of critically ill patients from the field. They are effective in reducing adverse events [23]. There is ample data on the effectiveness of MCCUs in high-income areas [23, 24]. A well-organized ambulance system in LMICs successfully reduces deaths [14, 22]. Observational studies have shown a decreased risk of acute events when transporting patients using a physician and nurse [25]. MCCUs have been deployed in war zones such as Ukraine [26], and there is at least one report from Hyderabad, India, of an MCCU deployed from a bus during COVID [27]. Below, we discuss this model within the structure, space, stuff, and staff paradigm.

Structure: The MCCU aims not to replace existing EMS systems but to enhance them. The ALS ambulance would always be the first point of contact for critically ill patients. Therefore, it is essential to have a working EMS system with reliable ALS services before adding on mobile critical care units. If reliable ALS ambulances are unavailable, then that should be addressed first.

For critically ill patients requiring more support and/or prolonged transport time, the ALS team can rendezvous with and transfer care to the MCCU. For example, in a patient with a snakebite, the ALS ambulance would take the patient to the nearest center where the patient could receive anti-snake venom and monitoring. But if the hospital is several hours away or the patient is deteriorating, requiring airway management and urgent anti-snake venom with monitoring for anaphylaxis, the MCCU could be called in to address urgent management while transporting the patient to the nearest hospital.

MCCUs must not be used as a substitute for primary care or to bypass readily available services at local healthcare centers. However, if that is not possible, then the MCCU should be able to care for the patient for a few hours.

Coupling this system with awareness and training of the local population on when and how to seek care is necessary and could significantly reduce mortality [22]. MCCUs could also be used for interhospital transfer between ICUs or from the emergency departments to the ICU when ALS ambulance transfer is not possible or risky.

Space: While MCCU can refer to large vehicles that need to be set up and must stay in place in the field, what I mention in this paper is truly mobile, based out of a van or a bus. MCCUs would be able to offer vastly expanded care to critically ill patients compared to the standard ALS transport that is often only comprised of paramedics; they also help reduce the incidence of adverse events during transport [23, 24].

Stuff: While MCCUs could manage certain critical care conditions entirely, as a destination ICU, the primary aim of the MCCU is to get the patient to a higher level of care. MCCU care should be kept to the shortest duration possible. Ideally, MCCUs are meant to be a bridge in providing critical care from when the patient needs care to when they are transferred to a higher level of care. They should be able to provide basic ICU care for a patient for several hours, allowing time for initial care and transport. This means having enough supplies, such as oxygen, mechanical ventilation, syringe pumps, medications, fuel, and batteries. The exact time the MCCU can support a patient would depend on local context and need.

Staff/social support: Staffing and training are possibly the most essential aspects of an MCCU. It is the crucial difference between an ALS ambulance and MCCU. It would be hard to justify staffing an MCCU with a physician in areas with resource limitations that often face a shortage of physicians. In most cases, the MCCU could be staffed by a trained nurse with a paramedic and a separate driver, and telehealth consultations could be conducted by a physician who could support multiple MCCUs in a region. This will likely achieve the same health outcomes if physicians staffed the MCCU [28]. However, care must be taken to appropriately train the nurses and EMTs in essential procedures and management. Where available, advanced practice providers (APPs) or equivalent would be invaluable in these situations. In areas where APPs do not exist, upskilling available healthcare workers through appropriate training channels would be essential. A good example is the development of the Emergency Medicine and Critical Care Clinical Officer Program (ECCCO) in Kenya [29]. The nurses, doctors, APPs, and paramedics staffing the MCCUs must be trained in triage and emergency/critical care and be knowledgeable in using the equipment on board and treatment protocols. Context-specific social support through community health workers, improving awareness, and gaining trust are essential for any project’s success.

## Conclusion

MCCUs enhance the emergency medical system in essential ways. When applied correctly, they will be crucial in addressing critical transportation delays and the time needed to receive appropriate lifesaving critical care in resource-limited settings. They are versatile and can be used to transfer severely ill patients to a higher level of care from the field, safely transfer critically ill patients between hospitals, and, sometimes, almost more importantly, provide standalone short-term critical care in regions where ICUs might be absent or immediately inaccessible. Incorporating care of the critically ill in this way will help patients when they need it most, improve access to timely critical care, reduce mortality, and help complement and strengthen existing systems of care.
